# Whole blood vs PBMC: compartmental differences in gene expression profiling exemplified in asthma

**DOI:** 10.1186/s13223-019-0382-x

**Published:** 2019-11-21

**Authors:** Daniel He, Chen Xi Yang, Basak Sahin, Amrit Singh, Casey P. Shannon, John-Paul Oliveria, Gail M. Gauvreau, Scott J. Tebbutt

**Affiliations:** 1 Centre for Heart Lung Innovation, University of British Columbia, Room 166, 1081 Burrard Street, Vancouver, BC V6Z1Y6 Canada; 2 Prevention of Organ Failure (PROOF) Centre of Excellence, Vancouver, BC V6Z2K5 Canada; 3 Department of Medicine, McMaster University, Hamilton, ON L8N3Z5 Canada; 4 Department of Pathology, Stanford University, Palo Alto, CA 94043 USA; 5 Department of Medicine (Division of Respiratory Medicine), University of British Columbia, Vancouver, BC V6Z1Y6 Canada

**Keywords:** Whole blood, PBMC, Gene expression, Asthma

## Abstract

**Background:**

Blood has proven to be a useful resource for molecular analysis in numerous biomedical studies, with peripheral blood mononuclear cells (PBMCs) and whole blood being the major specimen types. However, comparative analyses between these two major compartments (PBMCs and whole blood) are few and far between. In this study, we compared gene expression profiles of PBMCs and whole blood samples obtained from research subjects with or without mild allergic asthma.

**Methods:**

Whole blood (PAXgene) and PBMC samples were obtained from 5 mild allergic asthmatics and 5 healthy controls. RNA from both sample types was measured for expression of 730 immune-related genes using the NanoString nCounter platform.

**Results:**

We identified 64 uniquely expressed transcripts in whole blood that reflected a variety of innate, humoral, and adaptive immune processes, and 13 uniquely expressed transcripts in PBMCs which were representative of T-cell and monocyte-mediated processes. Furthermore, analysis of mild allergic asthmatics versus non-asthmatics revealed 47 differentially expressed transcripts in whole blood compared to 1 differentially expressed transcript in PBMCs (FDR < 0.25). Finally, through simultaneous measurement of PBMC proteins on the nCounter assay, we identified CD28 and OX40 (*TNFRSF4*), both of which are critical co-stimulatory molecules during T-cell activation, as significantly upregulated in asthmatics.

**Conclusions:**

Whole blood RNA preserved in PAXgene tubes is excellent for producing gene expression data with minimal variability and good sensitivity, suggesting its utility in multi-centre studies requiring measurement of blood gene expression.

## Background

Gene expression profiling is a fundamental feature of systems biology studies, which are ever-increasing in use to unravel the complexity of biological systems and their disease states. In particular, measurement of gene expression (‘transcriptomics’) in blood is an effective tool for the discovery of biomarkers of disease, and remains a key component of studies requiring large numbers of samples due to ease of collection and storage. Although the advent of single-cell RNA sequencing has made it possible to measure gene expression of specific blood cell types, there remain financial and technical barriers to its widespread use. Hence, the most common methods of blood RNA profiling either investigate RNA extracted from all blood cell types (i.e. whole blood) or only peripheral blood mononuclear cells (PBMCs). While PBMCs (lymphocytes and monocytes) play a significant role in the immune system, they remain a subset of all immune cells and do not include other cell types such as eosinophils and neutrophils. Thus, consideration of the compartment being investigated is required when carrying out blood gene expression studies, particularly with respect to the disease or other condition of interest.

Asthma is a chronic disease of the lungs that is characterized by frequent dyspnea, wheezing, coughing, and chest tightness due to inflammation of the airways. Asthma affects an estimated 334 million people worldwide and its prevalence continues to rise [1]. This is accompanied by a high economic burden, with recent estimates suggesting an annual cost (both from medical costs and loss of productivity) of €72.2 billion in the European Union [2] and $56 billion in the United States [3]. Asthma is a heterogeneous disorder with numerous immunological pathways contributing to pathogenesis. Historically, allergic asthma has been described as a T helper type 2 (T_H_2) cell-mediated inflammatory response, characterized by airway and blood eosinophilia. Further investigations have identified a subset of patients with a predominantly neutrophilic asthma characterized by involvement of T helper type 17 (T_H_17) cells. While asthma can be controlled with corticosteroid treatment, some patients are steroid-resistant and require specific targeted therapies to manage their symptoms. This is more common in those with T_H_17-predominant asthma [4].

The use of transcriptomics has been a key method in uncovering the phenotypes of asthma. Microarray analysis of airway epithelial brushings revealed two distinct clusters of T_H_2-high and T_H_2-low asthmatic patients, the latter of which displayed no significant difference in expression of T_H_2 cytokines (IL-4, IL-5, IL-13) when compared to healthy controls [5]. Although there is a systemic component to the pathophysiology of asthma, differentially expressed genes (DEGs) found in the blood of asthmatic individuals may not translate to those found in lung. Results from a recent U-BIOPRED study showed that DEGs associated with eosinophilic T_H_2 asthma in the lung were not found in whole blood; in fact, only one of the transcripts was detectable (SERPINB2) and not significantly different between asthmatics and healthy controls [6]. Similarly, investigations of PBMCs from asthmatic individuals also displayed a unique gene expression profile differing from that found in asthmatic lung as well as whole blood [7, 8]. Thus, in order to determine blood biomarkers of asthma, it is evident that the merits and limitations of investigating either blood compartment require thorough evaluation. In this study, we sought to compare the gene expression profiles of whole blood and PBMC compartments in a cohort of mild allergic asthmatic individuals and healthy controls.

## Methods

Whole blood (in PAXgene RNA tubes, PreAnalytiX, Hombrechtikon, Switzerland) and PBMCs (Ficoll preparation) were obtained from 8 mild allergic asthmatics defined as skin test positive to common aeroallergens and having FEV_1_ (forced expiratory volume in 1 s) ≤ 80% of predicted and methacholine PC_20_ (provocative concentration causing 20% fall in FEV_1_) ≤ 16 mg/ml, and 8 healthy controls (skin test negative, FEV_1_ ˃ 80% of predicted, methacholine PC_20_ ˃ 16 mg/ml) at McMaster University (Hamilton, ON, Canada) (Table 1). Prior to methacholine challenge, subjects were instructed to avoid the use of asthma medication prior to testing (for further details, see Ref. [9]). RNA was extracted from whole blood samples using PAXgene Blood miRNA Kit (PreAnalytiX, Hombrechtikon, Switzerland), then quantified (100 ng) for expression of 730 transcripts using the NanoString nCounter panCancer Immune Profiling panel (NanoString Technologies, Seattle, WA). PBMCs were lysed with RLT buffer (Qiagen, Hilden Germany) and analyzed for gene transcript and protein expression using the nCounter panCancer Immune Profiling plus Vantage 3D Protein Immune Cell Profiling panel (NanoString Technologies, Seattle, WA). In order to directly compare subject-specific pairs of whole blood and PBMCs we excluded data from 6 subjects (3 allergic asthmatic, 3 healthy controls) due to poor data quality of the PBMC sample as determined by NanoString quality control standard operating procedures (SOPs). Data were normalized using total sum scaling, and transcripts were filtered for low abundance using the geometric mean of negative controls (no target RNA). Normalization of protein data was also performed using total sum scaling, with an additional normalization against IgG negative controls. Differential expression testing between whole blood and PBMC fractions, as well as asthmatic and control groups, was performed using Linear Models for Microarrays and RNA-Seq (LIMMA, version 3.38.3) in the R statistical environment (version 3.5.2). Where appropriate, subjects were included into our linear modeling as random effects. False positives introduced by multiple testing were controlled for using the Benjamini–Hochberg false discovery rate, FDR (LIMMA default parameter). Gene expression data has been deposited in the Gene Expression Omnibus under accession number GSE132006, and normalized protein values can be found in Additional file 1.

**Table 1:** Characteristics of subjects analysed for both whole blood and PBMC gene expression

	Control	Asthma
Age (years)	26.6 ± 8.2	33.8 ± 15.6
Sex (female/male)	2/3	4/1
Atopic allergy	0	5
FEV_1_ (% predicted)	102.1 ± 13.0	90.2 ± 14.2
Methacholine PC_20_ (mg/ml)	˃ 16	2.1 (0.09–10.7)

Data are shown as mean ± standard deviation except for methacholine PC_20_ shown as geometric mean and range

## Results

**Fig. 1:**
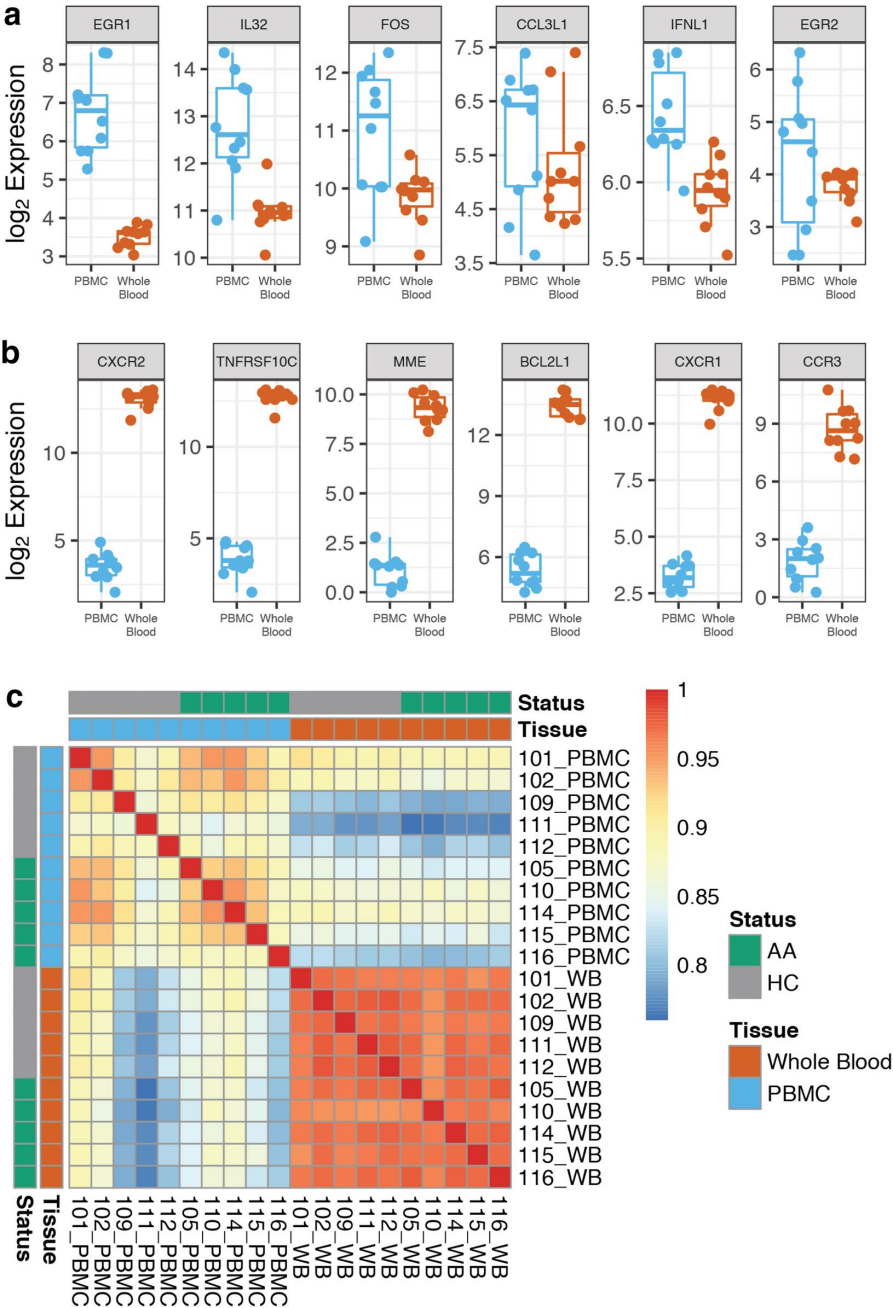
Comparison of gene expression profiles of PBMC and whole blood samples. Top 6 differentially expressed genes with increased expression (FDR < 0.25) in **a** PBMC samples compared to whole blood samples and **b** whole blood samples compared to PBMC samples. Y-axis values reflect log_2_-transformed gene expression. **c** Correlation matrix of gene expression measured in PBMC and whole blood samples

To examine overall gene expression, analysis of all measured transcripts on the NanoString Immune Profiling Panel (730 genes in total) revealed 704 differentially expressed genes (FDR < 0.25) between whole blood and PBMC compartments, of which only 6 genes (*EGR1*, *IL32*, *FOS*, *CCL3L1*, *IFNL1*, and *EGR2*) had increased levels of expression in PBMC samples compared to whole blood samples (Table 2; Fig. 1a, b). Pearson correlation analysis revealed an average correlation of 0.97 between whole blood samples and 0.90 between PBMC samples. Between whole blood and PBMC samples taken from the same subject, we calculated an average correlation of 0.87 (Table 3, Fig. 1c).

**Table 2:** Top 6 upregulated differentially expressed genes between PBMC and whole blood ordered by log_2_ fold change (log_2_FC)

PBMC			Whole blood		
Gene	log_2_FC	FDR	Gene	log_2_FC	FDR
EGR1	3.22	1.97×10^−11^	CXCR2	9.52	1.97×10^−24^
IL32	1.81	5.89×10^−6^	TNFRSF10C	8.89	5.89×10^−24^
FOS	1.11	2.65×10^−3^	MME	8.32	2.65×10^−22^
CCL3L1	0.54	2.17×10^−1^	BCL2L1	8.08	2.17×10^−23^
IFNL1	0.50	4.04×10^−2^	CXCR1	7.80	4.04×10^−24^
EGR2	0.49	2.01×10^−1^	CCR3	6.85	2.01×10^−17^

**Table 3:** Pearson correlation of RNA expression values between whole blood and PBMCs within subjects

Subject	Control					Asthma					Mean
	101	102	109	111	112	105	110	114	115	116	
Correlation	0.91	0.89	0.80	0.78	0.84	0.85	0.88	0.87	0.86	0.81	0.85

**Fig. 2:**
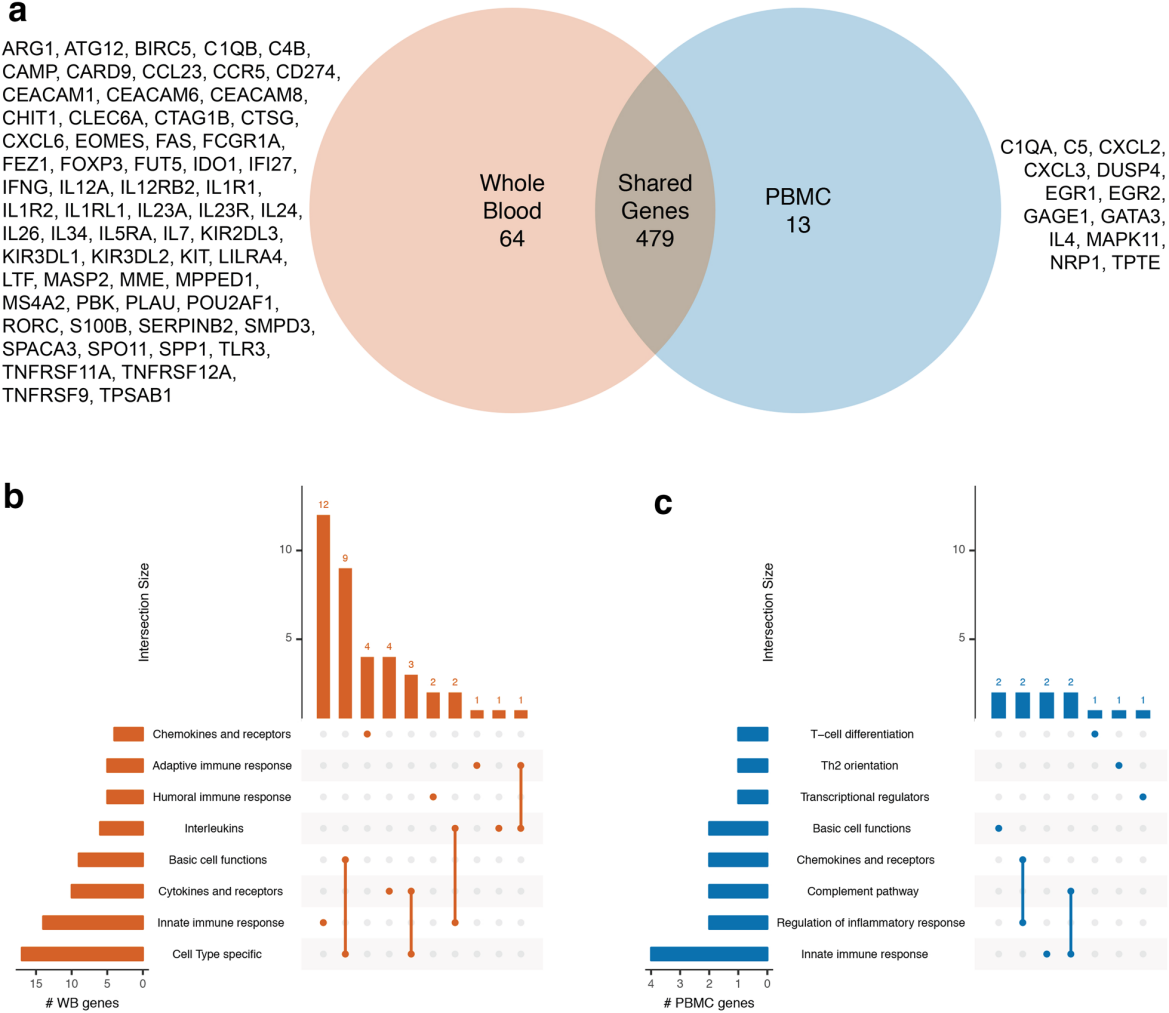
Detectable transcripts in whole blood and PBMC after removing lowly abundant transcripts. **a** Uniquely detected transcripts in whole blood versus PBMC. Intersection plots (generating using R package ‘UpSetR’ version 1.3.3) showing pathways associated with **b** unique whole blood transcripts and **c** unique PBMC transcripts. On the left are the number of uniquely detected whole blood (WB) or PBMC genes identified in each pathway. The matrix identifies gene intersections and combinations of intersections between pathways, with the top bar plot representing the number of genes within each association. Both intersection plots have been truncated to show the top 8 pathways by number of intersecting genes and a maximum of 10 between-pathway intersections

In order to examine compartment-specific pathways, we filtered for low abundant transcripts (all measured counts of a gene transcript falling below a background threshold set by the geometric mean of negative controls) and found 64 transcripts detected above background in whole blood but not PBMC samples, and 13 transcripts detected above background in PBMC but not whole blood samples (Fig. 2a). Notably, *GATA3* gene expression was detectable only in the PBMC samples along with *IL4*, both of which are mediators of T_H_2-immunity, in addition to other unique PBMC transcripts such as *CXCL2, CXCL3*, *EGR1*, and *EGR2*. We next analyzed tissue-specific genes using NanoString-curated transcript annotations. Unsurprisingly, the top pathways associated with unique transcripts in whole blood reflected a variety of immune processes such as the adaptive, innate, and humoral immune responses (Fig. 2b). For the PBMC-specific genes, the annotated pathways reflected T-lymphocyte and monocyte/macrophage-specific processes (Fig. 2c).

**Fig. 3:**
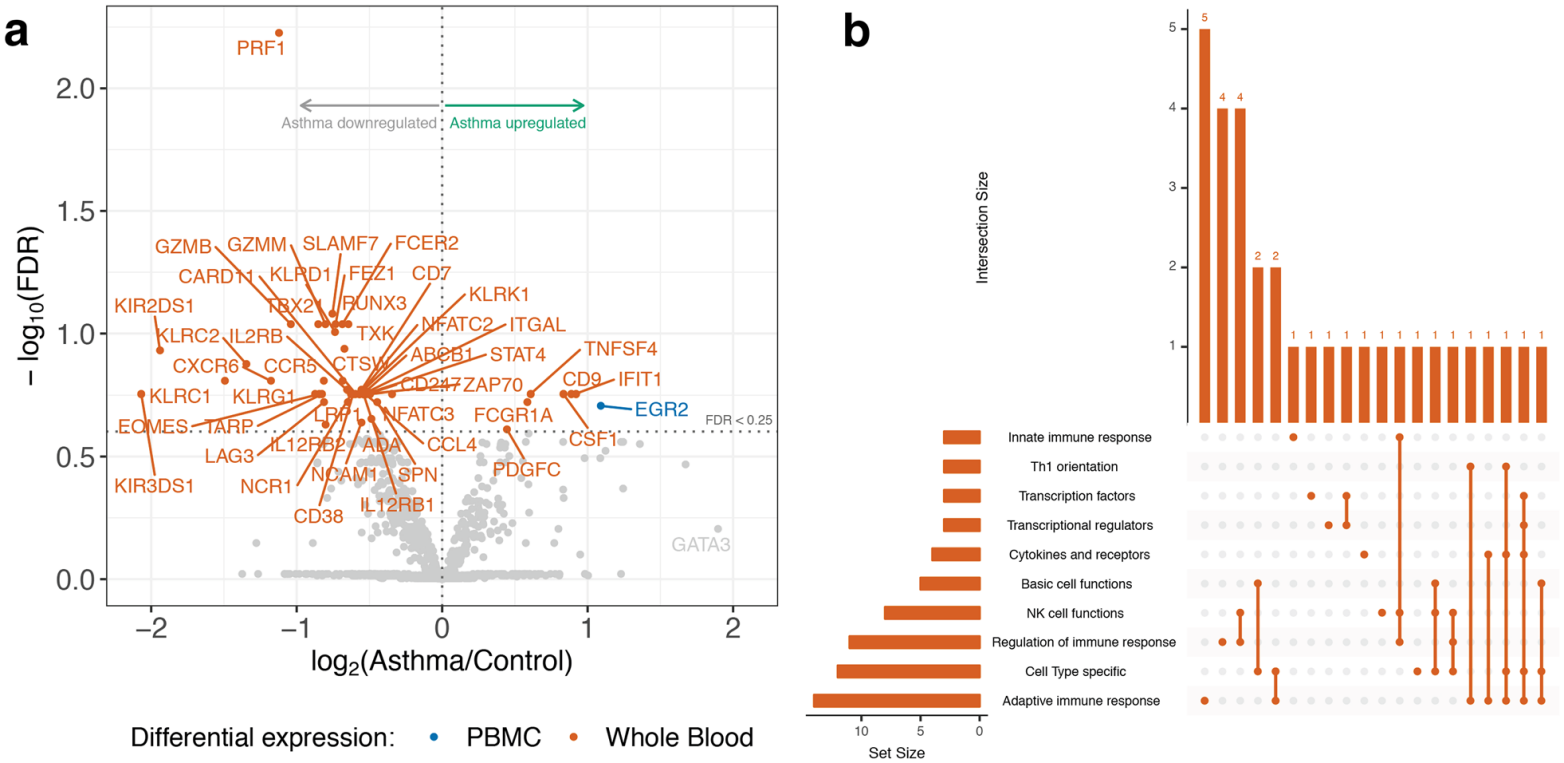
Differentially expressed genes in allergic asthma. **a** Volcano plot of differentially expressed genes (FDR < 0.25) in allergic asthmatics from whole blood and PBMC. **b** UpSet plot of downregulated genes in allergic asthma whole blood. Top 10 pathways by number of intersecting genes are shown, with a maximum of 20 between-pathway intersections

In addition to being uniquely detected in PBMC samples, *EGR2* was the only differentially expressed transcript (FDR < 0.25) when comparing asthmatics to healthy controls (see blue point in Fig. 3a, Additional file 2). Of note, *GATA3* was differentially expressed at a nominal *p*-value of 0.0028, but did not survive FDR cut-off. Compared to controls, we identified 41 downregulated and 6 upregulated differentially expressed transcripts (FDR < 0.25) in asthmatic whole blood. Pathway analysis of the downregulated transcripts reflected general immune processes in addition to specific functions relating to NK cells and T_H_1-mediated immunity (Fig. 3b).

**Fig. 4:**
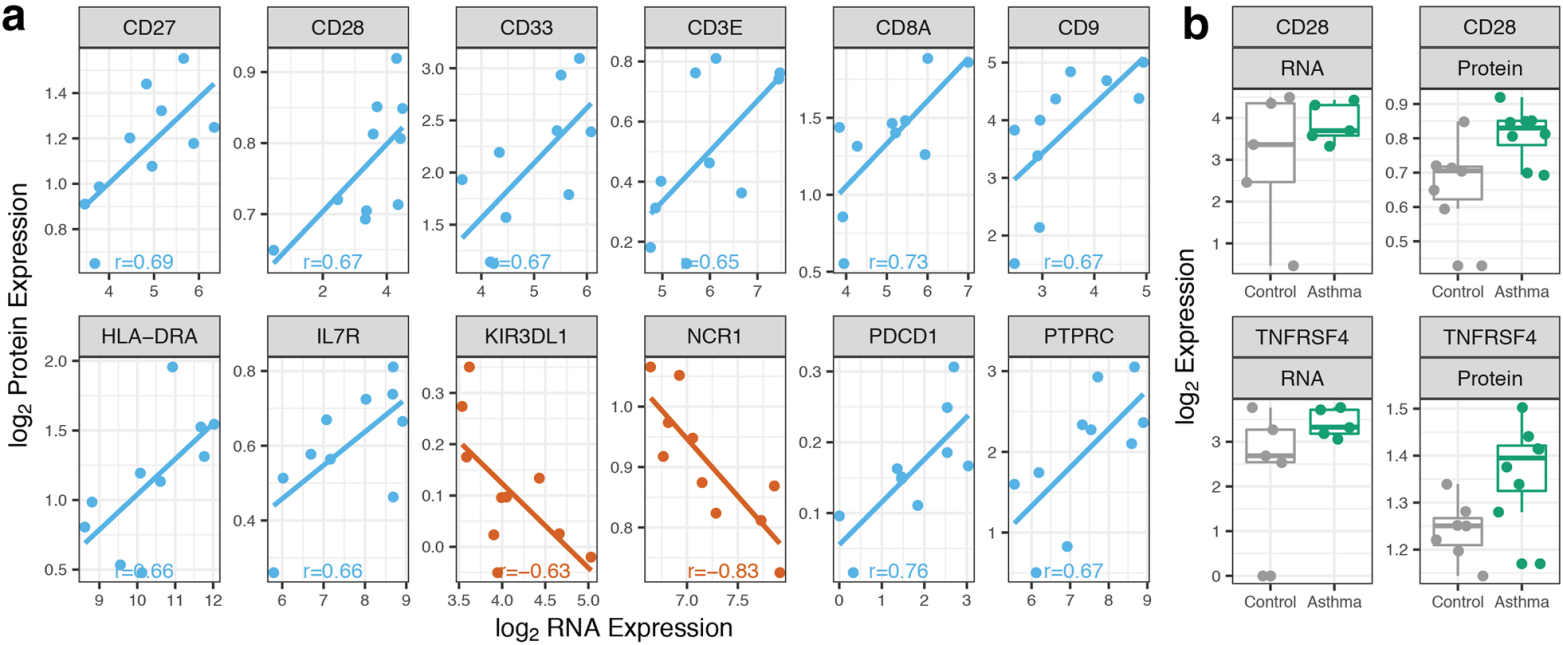
Analysis of protein expression in PBMCs. **a** Significant correlations (*p* < 0.05) of gene and protein expression in PBMCs (blue) and whole blood (orange), as measured using Pearson correlation. **b** Scatterplots of the two differentially expressed proteins in the allergic asthmatic cohort and their corresponding RNA scatterplots

The NanoString nCounter Vantage 3D Immune Cell Profiling assay allows for simultaneous measurement of RNA and protein expression. Of the 30 proteins on the panel, 10 were identified as positively correlated (*p* < 0.05, Pearson) with RNA expression levels measured in PBMCs (Additional file 3, Fig. 4a). NCR1 (natural cytotoxicity triggering receptor 1) protein expression was found to be significantly negatively correlated (*p* < 0.05, Pearson) with whole blood gene expression of NCR1 (Additional file 3, Fig. 4a). Differential expression analysis of measured proteins in all PBMC samples (8 asthmatic, 7 controls) revealed upregulated OX40 (*TNFRSF4*) and CD28 in the asthmatic cohort (Table 4, Fig. 4b); neither of these were differentially expressed in PBMC-extracted RNA, but the gene encoding for the OX40 ligand (*TNFSF4*) was upregulated in asthmatic whole blood-extracted RNA (Fig. 3a).

**Table 4:** Differentially expressed proteins between allergic asthmatics (n = 8) and healthy controls (n = 7)

Protein	log_2_FC	*p*-value	FDR
OX40	0.126	0.02	0.35
CD28	0.144	0.02	0.35

## Discussion

Unraveling the complexity of human disease has been enhanced with the rapid development of high-throughput molecular profiling. The examination of immune cells within blood samples is a commonly used method to elucidate disease mechanisms [10]. Here, we compared two types of blood sampling commonly used in immunological studies: PBMCs and whole blood.

Our results show that RNA extracted from whole blood stored in PAXgene tubes yielded higher counts of the 730 measured transcripts compared to RNA from PBMC lysates. Only 6 transcripts were expressed in greater quantities in PBMC lysates compared to whole blood, whereas whole blood had 704 transcripts with higher expression. A reason for this may be the lack of neutrophils, basophils, and eosinophils in PBMC samples. Basophils and eosinophils are only a small subset of all immune cells (0–2% and 1–7%, respectively). Neutrophils make up a majority of circulating nucleated blood cells (45–75%), but a relatively low RNA content relative to normal cells [11]. While this may be partially mitigated through overrepresentation of genes important in immune functions within our 730 measured transcripts (almost half of the 64 uniquely expressed transcripts we identified in whole blood are expressed by neutrophils), the strong correlation observed between PBMC and whole blood samples from the same subject (mean 0.87) suggests that granulocytic involvement is unlikely to explain the higher signal obtained from whole blood. We also cannot rule out technical differences in sample processing. Namely, there may have been disparities in the quality of total RNA extracted from the PAXgene RNA preservation reagent compared to the RLT buffer used for PBMC lysates. Within sample types, whole blood samples (r = 0.97) appear to be more robust than PBMC samples (r = 0.90), though both are acceptable as high positive correlations [12].

The data generated from our study present a contrast to previous studies comparing gene expression profiles obtained from PBMCs and whole blood. Feezor et al., Debey et al., Palmer et al., Bondar et al., Min et al. [13–17] found that RNA extracted from PBMCs had a higher abundance of gene expression compared to whole blood (PAXgene) as measured through microarray technology. This is likely due to the abundance of globin transcripts present in whole blood (80–90%), which causes decreased sensitivity in detection of other transcripts [14]. Hemoglobin was identified in a majority of these studies as being highly expressed in the whole blood gene expression profiles, and four of the five studies acknowledged globin as a potential cause of low signal-to-noise ratios in whole blood. Our targeted measurement approach to gene expression (730 genes), though more limited in scope, mitigates these effects as globin transcripts were not measured on the NanoString panCancer Immune Profiling Panel. Despite this, correlations between expression profiles of PBMC and whole blood were found to be between 0.78 and 0.91, which are in line with our results [13, 16].

Interestingly, another group used a similar approach to the aforementioned studies, yet found that whole blood (PAXgene) transcripts produced a greater signal compared to PBMC transcripts despite globin (*HBB*) being highly expressed [18]. A reason for this may be the use of the Affymetrix Human Exon (HuEx) 1.0 ST microarray, which measures gene expression through exon probing and is unique when compared to the aforementioned studies which used the U133A (Affymetrix, Santa Clara, CA), Lymphochip, HumanHT-12 v4 (Illumina Inc, San Diego, CA), and Sentrix Human-6 v2 (Illumina) microarrays. While the HuEx technology is able to discern alternatively spliced transcripts and thus gene isoforms, it has worse detection rates compared to conventional microarrays and has poorer reproducibility for genes with fewer exons [19]. With this in mind, it is possible that the effects of globin were dampened as the globin genes *HBA1*, *HBA2*, *HBB*, and *HBD* each contain 3 exons, which is low when compared to the mean number of exons per gene (8.8) [20].

The upregulated transcripts in PBMC samples (*EGR1*, *IL32*, *FOS*, *CCL3L1*, *IFNL1*, and *EGR2*) have distinct roles in lymphocytes and monocytes. *FOS*, which encodes for the proto-oncogene c-Fos, acts as a suppressor of the immune system [21]. IL-32 is primarily expressed in lymphocytes, monocytes, and natural killer cells, and induces production of other pro-inflammatory cytokines such as IL-8 and TNF-α [22]. *IFNL1* encodes for interferon lambda (IFN-λ, also known as IL-29), which is produced in response to viral infections by myeloid cells [23]. CCL3L1 is produced by PBMCs as a monocyte chemoattractant [24].

The early growth response transcription factors EGR1 and EGR2 play opposing roles in immunity; while EGR1 positively regulates T and B lymphocyte activation upon antigenic stimulation, EGR2 is involved in T cell anergy and is involved in the FasL-mediated apoptotic pathway [25]. In our analysis, we found that both *EGR1* and *EGR2* were only detected in PBMC samples, and *EGR2* was differentially expressed in our asthmatic cohort. EGR2 has been shown to be a crucial mediator of allergic asthma, as its expression is necessary in mast cells to direct CD4^+^ T cell migration to inflamed lung [26]. Though EGR2 negatively regulates T-cell activation, it is required for T_H_1, T_H_2, T_H_9, T_H_17, and cytotoxic T lymphocyte (CTL) differentiation and cytokine production [27]. T_H_2-mediated immunity was also represented in the uniquely expressed PBMC transcripts, as the GATA3 transcription factor and IL-4 cytokine were detected in PBMC but not whole blood samples, and both are crucial in the differentiation of naïve helper T cells towards a T_H_2 orientation.

Many of the statistically significant transcripts in the asthma whole blood cohort were related to cytotoxicity and natural killer (NK) cells. Differentiation of cytotoxic T-lymphocytes (CTLs) requires TBX21, RUNX3, EOMES, and STAT4 [28], all of which are associated with T_H_1-mediated immunity and were significantly downregulated in addition to the NK surface markers KIR2DS1, KIR3DS1, KLRC1, KLRD1, KLRG1, KLRK1. Transcripts encoding for cytolytic proteins such as PRF1, GZMB, and GZMM were all found to be significantly downregulated in the asthmatic cohort. Although CTLs and NK cells are both mononuclear cells, the differential expression of these transcripts was not detected in PBMCs. CD9 was also upregulated, and it has been used as a surface marker of eosinophils in asthma [29]. Overall, the differentially expressed transcripts found in whole blood describe a downregulation of T_H_1 differentiation and cell-mediated immunity. For a list of gene/protein abbreviations and their official HUGO names, please see Additional file 4.

The squared correlation coefficient of RNA transcript expression to protein expression has been reported to be ~ 0.4, meaning that mRNA levels account for roughly 40% of the variation in protein concentrations [30]. In our 30 measured proteins extracted from PBMCs, we calculated a squared coefficient of correlation of 0.12 between gene transcripts and proteins. However, we identified 12 proteins with strong correlations to their RNA abundance, which is consistent with a gene-specific level of protein expression [31].

The analysis of differential gene and protein expression identified an interesting insight into the pathophysiology of asthma. The two differentially expressed proteins in our asthmatic cohort were CD28 and OX40 (*TNFRSF4*), both of which are critical co-stimulatory molecules during T-cell activation. CD28 knockout mice do not develop an inflammatory response during allergen challenge [32], and OX40 is essential for optimizing CD4 and CD8 T-cell responses upon activation [33]. In our gene expression data, the ligand for OX40 (OX40L, *TNFSF4*) was also significantly upregulated in the whole blood samples of our asthmatic cohort. The OX40/OX40L axis is a central player in promoting T_H_2 polarization of naïve T-cells in the lymph node, with knockout mouse models of either molecule showing a marked reduction in airway hyperresponsiveness, eosinophilia, and pulmonary inflammation when challenged with ovalbumin [34, 35]. OX40 is expressed on T-cells, but can also be found on NK cells, NKT cells, and neutrophils [36], whereas OX40L is primarily expressed on antigen presenting cells such as B cells, macrophages, and dendritic cells but can be induced on other cell types such as mast cells [37], basophils [38], NK cells [39], and neutrophils [40]. Given the predominant role of T_H_2 cells in the mechanisms of asthma, it is no surprise that its activator OX40 is found to be upregulated in PBMCs of asthmatics and that widespread OX40L gene expression from a variety of cell types can be detected in whole blood, suggesting a consistently upregulated phenotype of T-cell activation.

## Conclusion

In summary, we show here that PAXgene-preserved whole blood is excellent for producing gene expression data with minimal variability and good sensitivity compared to PBMC samples. Despite the small sample size of our study, our paired analysis of whole blood and PBMC samples increased our power to detect differences. Studies requiring blood RNA samples across multiple sites should consider RNA from whole blood instead of PBMC due to its ease of processing (obtained directly from blood draw vs Ficoll isolation) and storage. Although we were limited by the number of transcripts examined, our gene panel did not contain globin genes and thus our data was not hindered by the suppressive effects of globin on the measurement of other transcripts. An added benefit of utilizing the NanoString platform is its precision and robustness; previous studies examining its reproducibility have found that it is comparable to quantitative PCR (qPCR) and exceeds that of RNA-seq and microarrays [41–43]. For the first time, we have shown gene expression profiling differences between blood compartments when comparing asthmatic subjects with non-asthmatic healthy controls. Furthermore, in whole blood transcript and PBMC protein expression, we identified the upregulation of the OX40/OX40L axis in the peripheral blood of asthmatic individuals, which has previously been identified as upregulated in serum of pediatric asthmatic patients [44, 45] and investigated as a potential therapeutic target in asthma [46].

## Supplementary information

**Additional file 1.** Normalized protein data obtained from healthy controls (HC) and allergic asthmatics (AA). Raw protein values were normalized using total sum scaling and IgG negative controls.

**Additional file 2.** Differentially expressed genes between allergic asthmatics and healthy controls in PBMC and whole blood samples. Comparison of gene expression between asthmatics and healthy controls using linear modeling (FDR cutoff set at 0.25).

**Additional file 3.** Spearman correlation of RNA expression values measured in whole blood and PBMC with measured protein expression in PBMCs. Correlation analysis of RNA expression values measured in whole blood and PBMC samples with protein expression values in PBMCs. Bold indicates significant correlation (*p* < 0.05).

**Additional file 4.** List of official HUGO gene/protein names. Table of gene/protein abbreviations with their corresponding official Human Gene Organization (HUGO) names.

## Data Availability

The gene expression dataset supporting the conclusions of this article is available in the Gene Expression Omnibus (GEO), GSE132006 (https://www.ncbi.nlm.nih.gov/geo/query/acc.cgi?acc=GSE132006), while normalized protein data is included within the article (Additional file 1).

## Abbreviations

DEG: differentially expressed gene
FDR: false discovery rate
FEV_1_: forced expiratory volume, 1 s
PBMC: peripheral blood mononuclear cell
PC_20_: provocative concentration for 20% fall in FEV_1_
PCR: polymerase chain reaction
qPCR: quantitative polymerase chain reaction
SOP: standard operating procedure
U-BIOPRED: Unbiased BIOmarkers in PREDiction of respiratory disease outcomes
