# Supplementary material for: Whole blood vs PBMC: compartmental differences in gene expression profiling exemplified in asthma
Source: Allergy Asthma Clin Immunol. 2019 Nov 21;15:67. doi: 10.1186/s13223-019-0382-x (PMC6873413; doi:10.1186/s13223-019-0382-x)
Supplement: Supplementary file 2 — Additional file 2. Differentially expressed genes between allergic asthmatics and healthy controls in PBMC and whole blood samples. Comparison of gene expression between asthmatics and healthy controls using linear modeling (FDR cutoff set at 0.25). [file 13223_2019_382_MOESM2_ESM.docx]

Additional File 2. Differentially expressed genes between allergic asthmatics and healthy controls in PBMC and whole blood samples.

| **Tissue** | **Gene** | **log_2_FC** | **FDR** |
| --- | --- | --- | --- |
| Whole Blood | PRF1 | -1.12 | 0.01 |
| Whole Blood | SLAMF7 | -0.75 | 0.08 |
| Whole Blood | GZMM | -0.80 | 0.09 |
| Whole Blood | FEZ1 | -0.73 | 0.09 |
| Whole Blood | FCER2 | -0.69 | 0.09 |
| Whole Blood | TBX21 | -0.85 | 0.09 |
| Whole Blood | RUNX3 | -0.64 | 0.09 |
| Whole Blood | GZMB | -1.04 | 0.09 |
| Whole Blood | KLRD1 | -0.74 | 0.10 |
| Whole Blood | TXK | -0.67 | 0.12 |
| Whole Blood | KIR2DS1 | -1.94 | 0.12 |
| Whole Blood | KLRC2 | -1.35 | 0.13 |
| Whole Blood | KLRC1 | -1.49 | 0.16 |
| Whole Blood | CTSW | -0.68 | 0.16 |
| Whole Blood | CCR5 | -0.81 | 0.16 |
| Whole Blood | CXCR6 | -1.18 | 0.16 |
| Whole Blood | IL2RB | -0.65 | 0.17 |
| Whole Blood | CD7 | -0.55 | 0.17 |
| Whole Blood | ITGAL | -0.51 | 0.17 |
| Whole Blood | KIR3DS1 | -2.07 | 0.18 |
| Whole Blood | CARD11 | -0.60 | 0.18 |
| Whole Blood | CD9 | 0.89 | 0.18 |
| Whole Blood | KLRK1 | -0.57 | 0.18 |
| Whole Blood | CD247 | -0.50 | 0.18 |
| Whole Blood | ABCB1 | -0.54 | 0.18 |
| Whole Blood | IL12RB1 | -0.54 | 0.18 |
| Whole Blood | KLRG1 | -0.87 | 0.18 |
| Whole Blood | ZAP70 | -0.49 | 0.18 |
| Whole Blood | EOMES | -0.84 | 0.18 |
| Whole Blood | TARP | -0.83 | 0.18 |
| Whole Blood | TNFSF4 | 0.61 | 0.18 |
| Whole Blood | NFATC2 | -0.57 | 0.18 |
| Whole Blood | CSF1 | 0.83 | 0.18 |
| Whole Blood | CCL4 | -0.53 | 0.18 |
| Whole Blood | CD38 | -0.61 | 0.18 |
| Whole Blood | STAT4 | -0.52 | 0.18 |
| Whole Blood | NFATC3 | -0.34 | 0.18 |
| Whole Blood | IFIT1 | 0.92 | 0.18 |
| Whole Blood | NCR1 | -0.63 | 0.18 |
| Whole Blood | FCGR1A | 0.59 | 0.19 |
| Whole Blood | SPN | -0.45 | 0.19 |
| Whole Blood | LRP1 | -0.65 | 0.19 |
| Whole Blood | LAG3 | -0.81 | 0.19 |
| PBMC | EGR2 | 1.09 | 0.20 |
| Whole Blood | ADA | -0.49 | 0.22 |
| Whole Blood | NCAM1 | -0.55 | 0.23 |
| Whole Blood | IL12RB2 | -0.80 | 0.23 |
| Whole Blood | PDGFC | 0.45 | 0.24 |
