# Supplementary material for: Whole blood vs PBMC: compartmental differences in gene expression profiling exemplified in asthma
Source: Allergy Asthma Clin Immunol. 2019 Nov 21;15:67. doi: 10.1186/s13223-019-0382-x (PMC6873413; doi:10.1186/s13223-019-0382-x)
Supplement: Supplementary file 3 — Additional file 3. Spearman correlation of RNA expression values measured in whole blood and PBMC with measured protein expression in PBMCs. Correlation analysis of RNA expression values measured in whole blood and PBMC samples with protein expression values in PBMCs. Bold indicates significant correlation (p < 0.05). [file 13223_2019_382_MOESM3_ESM.docx]

Additional File 3. Spearman correlation of RNA expression values measured in whole blood and PBMC with measured protein expression in PBMCs.

|  | PBMC | | Whole Blood | |
| --- | --- | --- | --- | --- |
| Gene | **Correlation** | ***p*-value** | **Correlation** | ***p*-value** |
| BTLA | 0.47 | 0.173 | 0.00 | 0.997 |
| CD14 | 0.58 | 0.076 | 0.48 | 0.164 |
| CD163 | 0.25 | 0.491 | -0.26 | 0.474 |
| CD19 | 0.45 | 0.191 | -0.14 | 0.692 |
| CD27 | **0.69** | **0.026** | -0.18 | 0.623 |
| CD274 | -0.16 | 0.663 | -0.11 | 0.767 |
| CD28 | **0.67** | **0.035** | 0.33 | 0.358 |
| CD33 | **0.67** | **0.035** | 0.13 | 0.718 |
| CD3E | **0.65** | **0.043** | 0.07 | 0.841 |
| CD4 | 0.30 | 0.404 | 0.37 | 0.297 |
| CD40 | 0.59 | 0.069 | 0.09 | 0.805 |
| CD40LG | -0.25 | 0.483 | 0.13 | 0.715 |
| CD68 | -0.58 | 0.078 | -0.48 | 0.156 |
| CD8A | **0.73** | **0.016** | 0.04 | 0.908 |
| CD9 | **0.67** | **0.033** | 0.29 | 0.422 |
| CTLA4 | -0.40 | 0.253 | -0.21 | 0.554 |
| HLA-DRA | **0.66** | **0.039** | 0.44 | 0.205 |
| ICOS | 0.30 | 0.402 | -0.17 | 0.647 |
| IL2RA | 0.15 | 0.673 | -0.48 | 0.163 |
| IL7R | 0.66 | 0.039 | -0.25 | 0.487 |
| KIR3DL1 | -0.26 | 0.473 | **-0.63** | **0.050** |
| NCAM1 | 0.20 | 0.576 | 0.10 | 0.784 |
| NCR1 | -0.04 | 0.898 | **-0.83** | **0.003** |
| NT5E | 0.56 | 0.091 | 0.36 | 0.308 |
| PDCD1 | **0.76** | **0.010** | -0.26 | 0.467 |
| PDCD1LG2 | 0.07 | 0.832 | 0.15 | 0.678 |
| PTPRC | **0.67** | **0.032** | 0.28 | 0.426 |
| TNFRSF18 | 0.40 | 0.279 | 0.33 | 0.347 |
| TNFRSF4 | -0.18 | 0.620 | -0.54 | 0.106 |
| TNFRSF9 | -0.22 | 0.547 | 0.05 | 0.888 |
| mean *r* | 0.347 |  | -0.046 |  |
| *r*^2^ | 0.120 |  | 0.002 |  |
