# Supplementary material for: Whole blood vs PBMC: compartmental differences in gene expression profiling exemplified in asthma
Source: Allergy Asthma Clin Immunol. 2019 Nov 21;15:67. doi: 10.1186/s13223-019-0382-x (PMC6873413; doi:10.1186/s13223-019-0382-x)
Supplement: Supplementary file 4 — Additional file 4. List of official HUGO gene/protein names. Table of gene/protein abbreviations with their corresponding official Human Gene Organization (HUGO) names. [file 13223_2019_382_MOESM4_ESM.docx]

Additional File 4. List of official HUGO gene/protein names.

| **Gene/Protein** | **HUGO Name** |
| --- | --- |
| BCL2L1 | Bcl-2-like protein 1 |
| CCL3L1 | C-C motif chemokine ligand 3 like 1 |
| CCR3 | C-C motif chemokine receptor type 3 |
| CXCL2 | C-X-C motif chemokine ligand 2 |
| CXCL3 | C-X-C motif chemokine ligand 3 |
| CXCR2 | C-X-C motif chemokine receptor 2 |
| EGR1 | early growth response 1 |
| EGR2 | early growth response 2 |
| EOMES | eomesodermin |
| FOS | Fos proto-oncogene |
| GATA3 | GATA binding protein 3 |
| GZMB | granzyme B |
| GZMM | granzyme M |
| HBA1 | hemoglobin subunit alpha 1 |
| HBA2 | hemoglobin subunit alpha 2 |
| HBB | hemoglobin subunit beta |
| HBD | hemoglobin subunit delta |
| IFNL1 | interferon lambda 1 |
| IL4 | interleukin 4 |
| IL4 | interleukin 5 |
| IL13 | interleukin 13 |
| IL32 | interleukin 32 |
| KIR2DS1 | killer cell immunoglobulin like receptor, two Ig domains and short cytoplasmic tail |
| KIR3DS1 | killer cell immunoglobulin like receptor, three Ig domains and short cytoplasmic tail |
| KLRC1 | killer cell lectin like receptor C1 |
| KLRD1 | killer cell lectin like receptor D1 |
| KLRG1 | killer cell lectin like receptor G1 |
| KLRK1 | killer cell lectin like receptor K1 |
| MME | membrane metalloendopeptidase |
| NCR1 | natural cytotoxicity triggering receptor 1 |
| PRF1 | perforin 1 |
| RUNX3 | RUNX family transcription factor 3 |
| SERPINB2 | serpin family B member 2 |
| STAT4 | signal transducer and activator of transcription 4 |
| TBX21 | T-box transcription factor 21 |
| TNFRSF10C | TNF receptor superfamily member 10c |
| TNFRSF4 | TNF receptor superfamily member 4 |
